# Critically Ill Obstetric Admissions to an Intensive Care Unit: A Prospective Analysis from a Tertiary Care University Hospital in South India

**DOI:** 10.5005/jp-journals-10071-237121

**Published:** 2019-02

**Authors:** Baby Sailaja K, Renuka MK

**Affiliations:** 1 Department of Critical Care Medicine, Sriramachandra Medical College and Research Institute, Chennai, Tamil Nadu, India; 2 Department of Anaesthesiology, Sriramachandra Medical College and Research Institute, Chennai, Tamil Nadu, India, e-mail: renuramanujam@gmail.com.

**Keywords:** Maternal outcome, Mechanical ventilation, Obstetric critical care, Postpartum, PRES

## Abstract

**Background:**

Critically ill obstetric patients represent a small proportion of intensive care unit (ICU) admissions. Physiological changes of pregnancy along with pregnancy specific diseases may lead to rapid deterioration of the health status of the parturient warranting ICU care. The present study aims to study the clinical profile and outcomes of the obstetric patients requiring ICU care.

**Study design and settings:**

Prospective observational study in the multidisciplinary ICU of a tertiary care teaching hospital conducted for a period of 2 years.

**Materials and methods:**

Demographic details, indication for ICU admission, severity of illness scores, interventions, complications and outcomes of the consecutive obstetric patients transferred to ICU were studied.

**Results:**

Ninety-one patients were admitted (26 per 1000 deliveries) to the ICU. Majority of them were postpartum (84.6%) and unbooked or referred (63.8%). Hypertensive disorders (24.2%) and obstetric hemorrhage (23.1%) were the major cause for admission to ICU. Forty three patients (47.3%) underwent cesarean delivery. Mechanical ventilation (54.9%), blood transfusion (46%), vasopressor therapy (22%) and dialysis (9.9%) were the various interventions provided in the ICU. Patients with sepsis had high mortality accounting for one third of ICU mortality. The ICU mortality rate was 9.9%.

**Conclusion:**

The present study showed a clinical profile and outcomes similar to the current scenario of critically ill obstetric patients nationwide. Further studies with a larger sample size may provide a better insight in this population.

**How to cite this article:**

Sailaja B, Renuka MK, *et al*. Critically Ill Obstetric Admissions to an Intensive Care Unit: A Prospective Analysis from a Tertiary Care University Hospital in South India. Indian J of Crit Care Med 2019;23(2):78-82.

## BACKGROUND

Maternal mortality refers to death due to complications of pregnancy and child birth. It reflects the quality of women's health care of a nation. The global maternal mortality rate (MMR) has declined by 44% over the last 25 years (1990-2015). The magnitude of reduction, however, shows a large discrepancy both within and between countries. The global MMR in 2015 was 216 deaths per 100000 live births. Almost 99% of these deaths occurred in developing countries ^1^. India recorded a MMR of 130 deaths per 100000 live births during this period^2^.

Young, healthy pregnant woman either exhibit a rapid worsening of their preexisting comorbidity because of present pregnant status or develop major complications without prior warning signs even leading to maternal death. The majority of such deaths can be prevented if these complications are managed with timely and effective obstetric critical care. There is a wide gap in the admission and mortality rate of obstetric patients admitted to the critical care units between developed and developing countries despite their similar clinical profile. The present study aims to analyse the incidence, clinical profile and outcomes of obstetric patients requiring admission to the multidisciplinary intensive care unit (ICU) of a tertiary care teaching hospital in South India.

## PROCEDURE

With the approval of institutional ethics committee, a prospective observational study was conducted for a 2-year period. All consecutive obstetric admissions (pregnant or within 6 weeks postpartum) to the multidisciplinary ICU during this period were enrolled into the study.

Patients data regarding age, gravida, parity, booking status, gestational age, preexisting diseases, medical conditions attributed by pregnancy, cause for ICU admission, parturient status on ICU admission, complications in ICU and interventions done were recorded. The severity of illness was assessed by admission day APACHE II (Acute Physiology And Chronic Health Evaluation II) and worst SOFA (Sequential Organ Failure Assessment) scores. The primary outcome was maternal mortality and ICU length of stay (LOS), and ventilator days were the secondary outcomes studied.

All statistical analyses were performed using Statistical Package for Social Science (SPSS, version 17) for Microsoft Windows. Descriptive statistics were presented as numbers and percentages. The data were expressed as mean and standard deviation. Independent sample student T test/Mann-Whitney test and Chi square test were applied for continuous variables and qualitative data respectively. Multiple Logistic Regression method was used wherever necessary and a *p* value <0.05 was considered statistically significant.

## RESULTS

A total of 91 obstetric patients were admitted to the ICU during the study period (Table 1). This accounted for 1.8% of the total admissions to the multidisciplinary ICU. The incidence of obstetric admissions to ICU was 2.6% (26 per 1000 deliveries) as there were 3,694 deliveries in the hospital during the study period. The ICU maternal death rate was 9.9 % (n = 9). The calculated MMR was 217 deaths per 100000 deliveries.

The mean age (years) of the study group was 29.52 ±5.9 with no significant difference among survivors (29.76 ±5.9) and non survivors (27.33 ±6.1). Thirty eight patients (41.8%) were referred from other hospitals and 20 (22%) patients did not undergo regular antenatal checkups. Fifty two (57.14%) patients had associated medical conditions, 63 (69.2%) patients were primipara and 77 patients (84.6%) were postpartum on transfer to the ICU. The mean APACHE II scores were significantly higher in non survivors compared to survivors [19.56±7.9 vs 10.17±7.1 (P =0.02)]. The worst SOFA was significantly higher in non survivors [(17.67±3.5 vs 4.97±3.7 (p = 0.00)] and maximum SOFA score observed was 22.

The booking status, presence of coexisting medical disorders and parity did not affect the maternal outcome. But patients admitted in antepartum state had a significantly higher mortality (*p* value 0.04) than postpartum patients.

**Graph 1:**
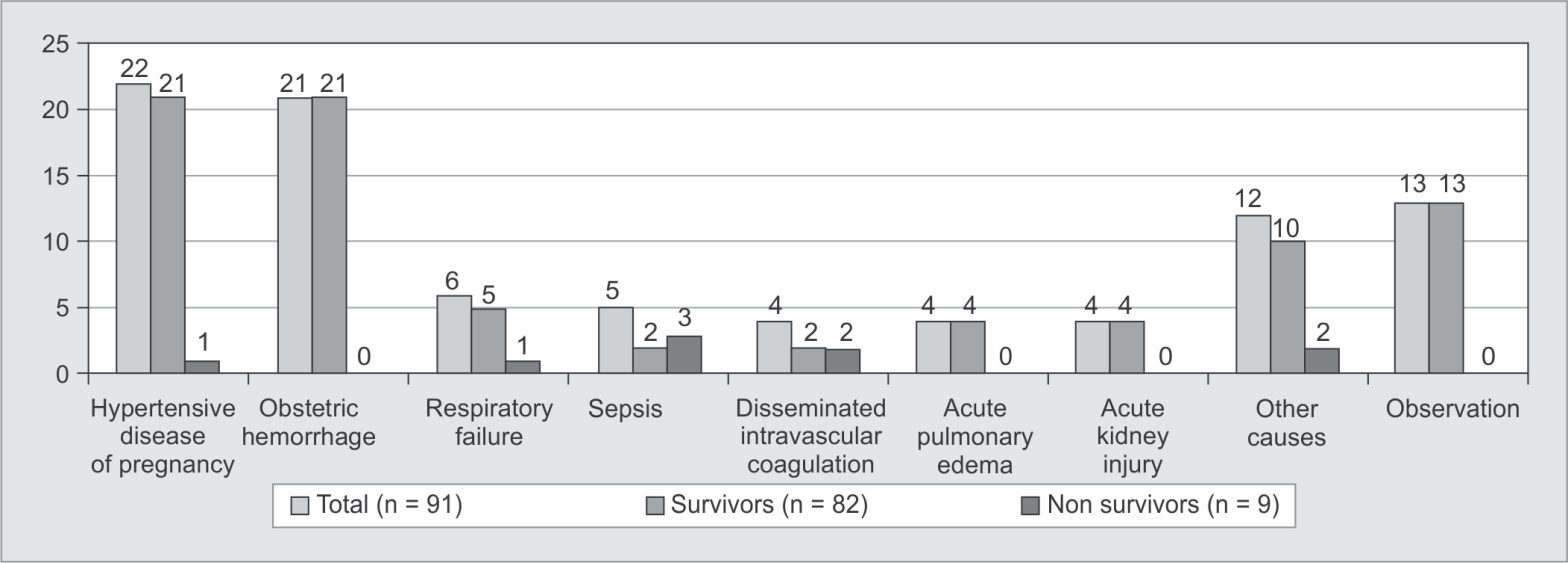
Indications for ICU admission of the study population

**Graph 2:**
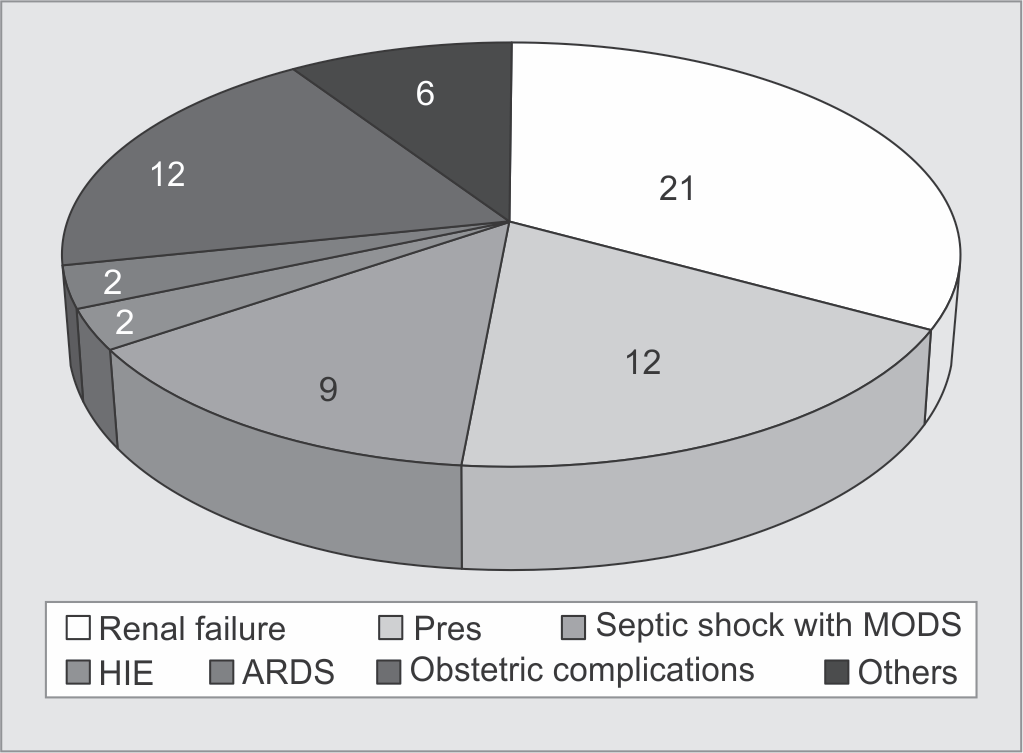
Major Complications in the patients admitted to ICU

The obstetric causes accounted for majority of ICU admissions (Graph 1) with hypertensive disorders [n = 22, (24.2%)] and obstetric hemorrhage [n = 21, (23.1%)] being the most common. Respiratory failure [n = 6, (6.6%)] was the commonest non obstetric cause for ICU transfer. Emergency cesarean section was done in 31 patients (34.1%) (Table 2). Mechanical ventilator support was provided to 50 patients (54.9%) for a mean duration of 2.72 days. Transfusions were needed in 42 patients (46.1%) and dialysis in nine patients (10.1%). Renal failure [n = 20 (22%)] and Posterior Reversible Encephalopathy Syndrome (PRES) [n = 12 (13%)] were the common complications observed in our patients (Graph 2).

## DISCUSSION

Obstetric patients are a clinically challenging group to any intensive care unit although they contribute only a small population. This was a prospective observational study conducted over a period of two years to analyse the obstetric admissions, their associated complications and interventions in the multidisciplinary ICU of a tertiary care teaching hospital.

The ICU utilization rate of 1.8% in our study is comparable with studies in the past as detailed in a systematic review by Pollock *et al*.^3^ The higher incidence of ICU admissions (2.6%) among obstetric patients in our study was consistent with studies by Harde *et al.* (2.8%),^4^ Bhadade *et al.* (2.8%)^5^ and Jain*et al.* (5.4%)^6^. However, data from previous studies^7–10^ did not show a similar high incidence. This may be due to differences in the criteria for ICU admission notably patients being transferred to the current facility due to lack of a dedicated obstetric high dependency unit in their hospital, patient referrals from other hospitals due to the severity of their illness and patients who were not booked developing complication subsequently.

**Table 1:** Demographic data of the obstetric patients admitted to the ICU during the study period

	*Total (n = 91)*	*Survivors (n = 82)*	*Non survivors (n = 9)*	*P value*
Age(years), mean± SD	29.52 ± 5.9	29.78 ± 5.9	27.33 ± 6.1	0.25
Parity [n, (%)]				0.71
< 2	63(69.2)	56(88.9)	7(11.1)	
≥ 2	28(30.8)	26(92.9)	2(7.1)	
Trimester [n, (%)]				0.27
I Trimester	5 (5.5)	5(100)	0(0)	
II Trimester	19(20.9)	15(78.9)	4(21)	
III Trimester	46(50.5)	42(91.3)	4(8.7)	
Postpartum	21(23.1)	20(95.2)	1(4.8)	
Prenatal care [n, (%)]				0.132
Booked	33(36.3)	32(97)	1(3)	
Unbooked	20(22)	16(80)	4(20)	
Referred	38(41.8)	34(89.5)	4(10.5)	
Parturient status [n, (%)]				0.004
Antepartum	14(15.4)	9(64.3)	5(35.7)	
Postpartum	77(84.6)	73(94.8)	4(5.2)	
Hospital LOS(Days), mean± SD	10.11 ± 5.7	10.06 ± 5.3	10.56 ± 9.0	0.59
ICU LOS(Days), mean± SD	3.04 ± 4	2.6 ± 3	7.0 ± 8	0.44
APACHE II, mean± SD	11.1 ± 7.6	10.17 ± 7.1	19.56 ± 7.9	0.002
Worst SOFA, mean± SD	6.2 ± 5.2	4.9 ± 3.7	17.6 ±3.5	0.000
Ventilator days, mean± SD	2.72±4.6	1.88 ± 3.41	6.51 ± 7.39	0.03
Vasopressor therapy [n, (%)]	20(21.9)	11(55)	9(45)	0.000
Days on vasopressors, mean± SD	3.30 ± 2.79	2 ± 1.18	4.89 ± 3.4	0.035

SD: Standard Deviation; LOS: length of stay; ICU: Intensive Care Unit; APACHE: Acute Physiology and Chronic Health Physiology; SOFA: Sequential Organ Failure Assessment;

**Table 2:** Obstetric and nonobstetric interventions among the study population

*Intervention*	*No of patients*	*Percentage (%)*
Emergency LSCS	31	34
Elective LSCS	12	13
Emergency laparotomy	13	14
Mechanical ventilation	50	55
Blood products transfusions	42	46
Vasopressors	20	22
Renal replacement therapy	9	10
Tracheostomy	4	4

LSCS: Lower segment cesarean section

Advanced maternal age did not carry an increased risk of maternal mortality in the present study population unlike that observed by Bhadade *et al*.^5^ The mean age of the study group with 23% in advanced maternal age (35 yrs and above) was comparable with recent studies^4,10–12^. Majority of our study population were primiparous (69.2%). Similar observations were made by Gombar *et al.* (54.3%)^7^ and Dasgupta *et al.* (58.3%)^8^. This is further explained by the higher incidence of PIH in our study population which is common in primipara.

There was a postpartum predominance comparable to other studies^7,9,12,13^. Postoperative transfer of patients with eclampsia and obstetric haemorrhage irrespective of their haemodynamic status and patients referred from other hospitals following a complication of delivery may explain this. Although the number of antepartum admissions was less and on par with the above studies, we observed a significant mortality among them. The mean hospital and ICU LOS (10.1 and 3.04 days respectively) of the study population were comparable to the recent studies^4,6,11,14,15^. But wide variations in the duration of ICU stay in both survivors and non survivors had been a confounding factor for this data. This may be the reason for the longer ICU LOS in non survivors (7days versus 2.6 days) not correlating with mortality like in other studies.

Hypertensive disorders followed by obstetric haemorrhage were the common causes for ICU transfer in the present study similar to other national^5–7^ and international studies^12,16^. There was an increase in the incidence of sepsis due to obstetric or non-obstetric causes in the recent studies − 11.4%^6^, 27.15%^7^, 13.17%^8^, 12.5%^10^. Our study observed only 5.5% of admissions to have sepsis. This was similar to other recent Indian studies^11,13,15^ although Harde *et al.*^4^ and Bhadade *et al.*^5^ had very low sepsis rate at admission.

The obstetric surgical interventions done were cesarean section in 47.3% and emergency laparotomy in 14.3% patients. Such high rate of cesarean section in patients requiring ICU care was common^3,8–10^. Mechanical ventilation, transfusion of blood products and initiation and maintenance of vasopressor support were the usual interventions done in our study. Mechanical ventilation was the most common intervention done in the ICU for obstetric patients as described in the systematic review by Pollock *et al.*^3^ [Developing countries-41% (range 3.0-100%) and Developed countries-41.5% (range 13.0-76.0%)] and in various recent studies^8,10,11^. In the present study, 54.9% of patients were mechanically ventilated. Eclampsia (13.2%) and obstetric haemorrhage (17.6%) were the most common indications for mechanical ventilation in our study. Seventy eight percent of these patients were already intubated in operating room or Emergency department. Only 22% of patients required invasive or noninvasive ventilation during the course of ICU stay. Two patients needed prone ventilation in view of acute respiratory distress syndrome (ARDS)^17^. The mean duration of ventilation was 2.7 days. Non survivors required a significantly longer period of ventilation, (6.5 days versus 1.9 days, p=0.03).

Blood products were transfused in the ICU in 42 patients (46%) while another 13 (14.3%) patients received transfusion even before transfer to ICU. The blood components therapy comprised packed cells in 48.3%, fresh frozen plasma in 38.5%, platelet concentrate in 33% and cryoprecipitate in 22% of patients. The transfusion rates were comparable with Harde *et al*.^4^, Sriram Robertson *et al*.^12^ and Karnad *et al*.^18^ There were no maternal deaths due to obstetric haemorrhage in our study primarily due to adequate and timely resuscitation with blood products. Our observations were consistent with the above studies^4,12^. Twenty patients (22%) had unstable haemodynamics requiring vasopressor support and they had a significantly high risk for mortality (p = 0.000). We also observed that higher doses and longer duration of vasopressor therapy were associated with adverse maternal outcome leading to death.

The incidence of acute renal failure requiring renal replacement therapy varied among recent studies^5,6,8,10,12,18^. In the present study 20 patients (22%) developed renal failure. Seventeen patients (18.7%) had developed acute kidney injury (AKI) as per Kidney Disease: Improving Global Outcomes (KDIGO)^19^ guidelines. Three patients with chronic kidney disease went in for acute deterioration. Though 12 patients (13.2%) required dialysis, three patients could not be dialysed because of severe haemodynamic instability. Six (6.6%) out of the nine patients who underwent dialysis died and three patients were discharged on dialysis.

The maternal mortality rate varied widely between developed and developing countries. It ranged between low or no maternal deaths^9,10,12,14,16^ in the former to as high as 41.67%^13^ in the later. The present study population had a mortality rate of 9.9%. Obstetric haemorrhage (27.1%) and hypertensive disorders (14.0%) are the most common causes of maternal mortality globally, followed by sepsis (10.7%)^20^. Though they together accounted for 57.3% of ICU transfers in our study there was only one maternal death due to these. However, we observed that sepsis due to obstetric or nonobstetric cause led to a significantly high mortality (60%) much higher than that observed by Gombar *et al*. (48.9%)^7^. Disseminated intravascular coagulation secondary to intrauterine death (IUD), viral pneumonia ARDS, peripartum cardiomyopathy and tuberculous meningitis were the other causes of death in this study. Among the survivors, nine patients suffered obstetric morbidity needing hysterectomy. Three patients were tracheostomised, one needed mechanical ventilation for more than 10 days following status epilepticus and two patients developed hypoxic ischemic encephalopathy secondary to cardiac arrest outside the ICU. Two patients with AKI became dialysis dependent.

Although the study analyzed various predictors of obstetric illness a multiple logistic regression analysis however found that none of the variables had an association as an independent risk factor for maternal mortality. Furthermore, the results of our study cannot be applied to the entire obstetric population of the nation. Lack of uniformity of health care services across the country and need for referrals to tertiary care hospitals leading to delay in providing treatment may adversely affect the maternal outcome.

## CONCLUSION

This study provides an insight into the characteristics of obstetric patients needing intensive care services in South India. However, given the limited sample size and period of study, we conclude that further research may help in achieving a more meaningful data of predictors to reduce maternal mortality and improve health care in this section of patients.
